# Association of estradiol and progesterone levels on human chorionic gonadotropin day with pregnancy outcomes in IVF/ICSI fresh embryo transfer

**DOI:** 10.3389/fendo.2026.1831029

**Published:** 2026-06-12

**Authors:** Qiaofei Tang, Honggan Yi, Yantian Zheng, Man Yang

**Affiliations:** 1 Reproductive Medicine Center, Meizhou People’s Hospital, Meizhou, China; 2 Meizhou Municipal Engineering and Technology Research Center for Molecular Diagnostics of Major Genetic Disorders, Meizhou People’s Hospital, Meizhou, China

**Keywords:** estradiol, *in vitro* fertilization, intracytoplasmic sperm injection, pregnancy outcome, progesterone

## Abstract

**Objective:**

This retrospective cohort study investigated the association between estradiol (E_2_), progesterone (P) levels on human chorionic gonadotropin (HCG) day and the pregnancy outcomes of fresh embryo transfer cycles in *In Vitro* Fertilization/Intracytoplasmic Sperm Injection (IVF/ICSI) procedures.

**Methods:**

A retrospective analysis was performed on 1520 cycles from October 2015 to December 2024. The patients were divided into the low and high hormone groups based on the E_2_ and P levels, and the differences of pregnancy outcomes were compared. Logistic regression analysis was used to investigate the influence of the E_2_ and P levels on the pregnancy outcomes adjusted for maternal age, paternal age, infertility type, infertility factors, fertilization method, COH protocol, forward motile sperm concentration, and ovarian hyperstimulation syndrome, embryo transfer time, and number of transferred embryos.

**Results:**

Among 1520 cycles, the clinical pregnancy rate was 45.0%, biochemical pregnancy rate 7.7%, and live birth rate 34.9%. The cutoff value of E_2_ was 2607.0 pg/mL, and P was 0.47 ng/mL based on Restricted Cubic Spline (RCS) analysis. Patients with E_2_ ≤2607.0 pg/mL and P ≤0.47 ng/mL had significantly higher clinical pregnancy rates but lower biochemical pregnancy rates than those with E_2_ >2607.0 pg/mL and P >0.47 ng/mL. Logistic regression analysis showed that E_2_ ≤2607.0 pg/mL (OR: 2.040, 95% CI: 1.627-2.558, *p* < 0.001), and P ≤0.47 ng/mL (OR: 1.970, 95% CI: 1.539-2.521, *p* < 0.001) were associated with higher clinical pregnancy rate, while E_2_ ≤2607.0 pg/mL (OR: 0.573, 95% CI: 0.387-0.848, *p* = 0.005), and P ≤0.47 ng/mL (OR: 0.455, 95% CI: 0.265-0.781, *p* = 0.004) were associated with lower biochemical pregnancy rate. The AUCs of E_2_ and P for predicting clinical pregnancy were 0.570 and 0.579, respectively.

**Conclusions:**

E_2_ and P levels on HCG day within the identified threshold range are related to improved clinical pregnancy and reduced biochemical pregnancy in IVF/ICSI fresh embryo transfer cycles.

## 1 Introduction

The World Health Organization (WHO) defines infertility as the inability of a couple to achieve conception following 12 months of regular sexual activity without the use of any contraceptive measures (1). Globally, the prevalence of infertility among the global reproductive-age population is showing a continuous upward trend (2, 3). In China, the infertility prevalence among reproductive-aged couples keeps rising and presents an obvious younger tendency (4, 5). Against the backdrop of the increasingly serious infertility problem worldwide, assisted reproductive technology (ART) has become a key means to improve the fertility predicament (6, 7). *In vitro* fertilization (IVF) mainly addresses issues such as blocked fallopian tubes and unexplained infertility in women, while intracytoplasmic sperm injection (ICSI) targets complex situations such as severe oligoasthenospermia or azoospermia (requiring sperm retrieval through biopsy) in men (8–10).

As of now, the overall pregnancy rate of fresh embryo transfer cycles remains at about 40%-55% (11, 12). Embryo implantation failure remains the core bottleneck that hinders the success rate of treatment (13). Estradiol (E_2_) regulates the proliferation of endometrial epithelial cells, glandular development, and angiogenesis, thereby establishing a basic physiological environment suitable for embryo implantation in the endometrium (14). Progesterone (P) induces the transformation of the endometrium from the proliferative phase to the secretory phase, regulates the opening time and functional state of the receptive window of the endometrium (15, 16). In IVF/ICSI cycles, human chorionic gonadotropin (HCG) triggers final oocyte maturation for oocyte retrieval (17, 18). Serum E2 and P levels on HCG day reflect ovarian response, correlate closely with endometrial receptivity, and profoundly affect embryo implantation outcomes (19, 20).

Existing studies have demonstrated conflicting effects of elevated HCG-day E_2_ levels on fresh embryo transfer outcomes. Excessively high E_2_ may suppress endometrial epithelial apoptosis and impair glandular differentiation, reducing endometrial receptivity and exacerbating embryo-endometrial asynchrony, particularly in ICSI cycles (21, 22). Conversely, elevated E_2_ may indicate favorable ovarian response, yielding more high-quality oocytes and indirectly improving pregnancy outcomes (23, 24). Even in the ICSI cycles, it can indirectly improve the pregnancy outcome by enhancing the quality of the embryos. Ochsenkühn et al. found that the increase in P on the HCG day shortens the opening time of the endometrial receptivity window and associated with unfavorable pregnancy outcomes (25). However, regarding the critical value of progesterone increase, the conclusions from different studies vary significantly (26). Although the associations of HCG-day E_2_ and P levels with IVF/ICSI pregnancy outcomes have been widely investigated, the findings remain controversial, with no unified optimal hormonal cutoff values. Most previous studies adopted linear analyses and failed to characterize the non-linear dose–response relationship between these hormones and pregnancy outcomes. The aim of this study is to explore the relationship between the E_2_ and P levels on the HCG day and the pregnancy outcomes of fresh embryo transfer cycles in IVF/ICSI procedures. In addition, Restricted Cubic Spline (RCS) analysis can accurately identify the inflection point of hormone levels, avoid bias caused by artificial grouping, and reliably determine the clinically optimal cutoff value, in order to provide a basis for optimizing the hormone monitoring strategies and transplantation decisions in ART treatment.

## 2 Materials and methods

### 2.1 Study cohort

It was a retrospective cohort study that consecutively included infertile patients who underwent IVF/ICSI fresh embryo transfer at the Reproductive Medicine Center of our hospital from October 2015 to December 2024 without selective exclusion. All inclusion and exclusion criteria were applied uniformly to minimize selection bias. This study is guided by the Declaration of Helsinki and approved by the Ethics Committee of Meizhou People’s Hospital.

Inclusion criteria (1): have clinically normal ovarian reserve function (①basal FSH <10 IU/L; ②antral follicle count (AFC) ≥5-7; ③anti-Müllerian hormone (AMH) ≥1.2 ng/mL) (27); (2) undergo controlled ovarian hyperstimulation (COH) and receive IVF/ICSI treatment; (3) transplant high-quality embryos (refers to embryos with normal morphology, uniform cell division and minimal fragment proportion that meet routine clinical transfer criteria) and receive standardized luteal support after transplantation (the luteal phase was defined based on the day of HCG administration (designated as day 0). Specifically, LH surge was monitored starting from approximately six days prior to the HCG trigger day in controlled ovarian hyperstimulation cycles. The luteal phase was considered to commence on the day of HCG trigger (day 0) when spontaneous LH surge was absent or suppressed); and (4) have complete clinical data, including hormone test results, follicular monitoring records, and pregnancy follow-up information.

Exclusion criteria: (1) severe internal or external diseases (such as dysfunction of heart, liver, kidney, or blood system diseases), organic lesions of the reproductive system (such as ovarian tumors, cervical insufficiency), or endometriosis-related diseases (such as endometriosis, adenomyosis); (2) previous history of repeated implantation failure or repeated spontaneous abortions; and (3) fetuses with major structural or chromosomal abnormalities diagnosed by prenatal examination. Patients with a history of previous IVF/ICSI cycles were not excluded, provided they met all other inclusion criteria.

To satisfy the statistical independence assumption, only one fresh embryo transfer cycle per patient was included in the present analysis. No patients contributed multiple treatment cycles to the cohort, so there was no within−patient correlation among observations. Accordingly, it was not necessary to apply generalized estimating equations (GEE), mixed−effects models, or cluster−robust standard errors to adjust for repeated measures.

### 2.2 Medical records and data collected

The electronic medical records of Reproductive Medicine Center served as the source for collecting information on parental characteristics and IVF/ICSI procedures. The associated factors of pregnancy outcomes in fresh embryo transfer in IVF/ICSI cycles investigated in this study were maternal age, paternal age, infertility types (primary infertility and secondary infertility), infertility factors (tubal factor, ovulatory dysfunction, and other factors), fertilization methods (IVF, ICSI, IVF+ICSI), COH protocol, forward motile sperm concentration after treatment, ovarian hyperstimulation syndrome (OHSS), embryo transfer time, number of transferred embryos, and hormone level on HCG day (E_2_ and P).

Maternal and paternal ages were each stratified into two subgroups: <35 years and ≥35 years. Based on pregnancy outcomes, participants were categorized into: clinical pregnancy, biochemical pregnancy, and live birth (28, 29). Clinical pregnancy is defined as a positive serum HCG on day 14 post-IVF/ICSI, combined with the presence of a gestational sac in the uterine cavity as indicated by vaginal ultrasound at 4 weeks post-IVF/ICSI. Biochemical pregnancy refers to an early pregnancy loss in which serum HCG is positive 14 days after embryo transfer, but no clinical pregnancy occurs subsequently, and serum HCG decreases to the normal range within a short period. Live birth refers to the outcome in which, after a confirmed clinical pregnancy, the pregnancy continues to 28 gestational weeks or more, resulting in the delivery of a live newborn with no fatal malformations and stable vital signs.

Primary infertility refers to the inability of a couple to achieve pregnancy after 12 months of regular, unprotected sexual intercourse, in cases where the female partner has no prior history of conception (30, 31). In contrast, secondary infertility is characterized by the couple’s difficulty in conceiving again, despite having experienced at least one successful clinical pregnancy before (31). Controlled ovarian hyperstimulation (COH) protocols were categorized into six subgroups, namely GnRH agonist (GnRH-a) long protocol, GnRH antagonist protocol, long term follicular protocol, GnRH-a short protocol, GnRH-a prolonged protocol, and other protocol. In this study, all patients underwent IVF/ICSI treatment. The choice of fertilization method was based on semen parameters and clinical indications, following the routine clinical practice of our reproductive center.

### 2.3 Exploration of the relationship between E_2_, P and pregnancy outcomes

Since there is currently no unified standard for E_2_, and P level based on clinical pregnancy outcome indicators (16, 32, 33), the optimal cutoff values for continuous variables were selected. In this study, the dose-response relationship between HCG daily E_2_, P and pregnancy outcomes was analyzed using restricted cubic spline (RCS) functions to determine the cut-off value (34–36). The skewed distributions of E_2_ and P were log-transformed, and continuous confounding variables were standardized. Then, a 4-node (based on 2.5th, 25th, 75th, and 97.5th percentiles) RCS regression model was constructed using the “rms” package in R software. The turning point where the slope of the curve changes significantly was identified from the dose-response curve as the potential cut-off value. After verifying the significance of the difference and assessing the clinical rationality through logistic regression analysis, the final cut-off value was determined. According to the cutoff values, the study subjects were divided into low E_2_ group (E_2_ ≤ cutoff value) and high E_2_ group (E_2_ > cutoff value); low P group (P ≤ cutoff value) and high P group (P > cutoff value).

### 2.4 Statistical analysis

Data analysis was conducted using SPSS 26.0 software. All variables had <5% missing values, which were handled by multiple imputation to avoid bias from complete−case analysis. Measurement data were expressed as mean ± standard deviation (`x± SD), and comparisons between groups were performed using t-tests or analysis of variance; count data were expressed as rates (%), and comparisons between groups were conducted using χ^2^ tests. The RCS model identified inflection points with significant nonlinearity for E_2_ and P. The subjects were grouped based on the cut-off values of E_2_ and P on the HCG day. Candidate cutoff values were verified by logistic regression to confirm statistical significance and clinical rationality. Multivariate logistic regression analysis was used to analyze the relationship between E_2_ and P levels and the pregnancy outcomes, such as clinical pregnancy, biochemical pregnancy, and live birth. Age, infertility type, infertility factors, fertilization methods, COH protocol, forward motile sperm concentration after treatment, and OHSS were adjusted as confounding factors. Odds ratios (OR) and 95% confidence intervals (CI) were calculated. Variables were selected based on clinical relevance and previous literature. All pre−specified confounders were included in the model using forced entry. Multicollinearity was assessed using variance inflation factor (VIF). All VIF values were < 2, indicating no significant multicollinearity. The accuracy of E_2_ and P in evaluation of pregnancy outcomes was evaluated by calculating the area under the ROC curve (AUC). *p* < 0.05 was considered statistically significant.

## 3 Results

### 3.1 Overall baseline characteristics of study cohort

In this study, among all cycles, 1009 (66.4%) were from mothers < 35 years old, and 511 (33.6%) were from mothers aged ≥ 35 years old; 793 (52.2%) cycles involved fathers < 35 years old, while 727 (47.8%) involved fathers aged ≥ 35 years old. Regarding infertility types, there were 704 (46.3%) cases of primary infertility and 816 (53.7%) cases of secondary infertility. In terms of treatment methods, there were 1233 (81.1%) patients underwent IVF, 272 (17.9%) patients underwent ICSI, and 15 (1.0%) underwent IVF+ICSI. 795 (52.3%), 441 (29.0%), 113 (7.4%), 120 (7.9%), and 29 (1.9%) patients performed GnRH-a long COH protocol, GnRH antagonist protocol, long term follicular protocol, GnRH-a short protocol, and GnRH-a prolonged protocol, respectively. 1444 cases (95.0%) underwent cleavage-stage embryo transfer, and 76 cases (5.0%) received blastocyst-stage embryo transfer. Among them, 1284 cases (18.7%), 1213 cases (79.8%) and 23 cases (1.5%) had 1, 2 and 3 embryos transferred respectively. There were 16 (1.1%) patients developed OHSS. In this study, 684 (45.0%) cycles achieved clinical pregnancy, 117 (7.7%) cycles resulted in biochemical pregnancy, and 531 (34.9%) cycles had live birth (**Table 1**).

**Table 1:** Overall baseline characteristics of study cohort.

Variables	Number (%)/mean ± SD
Number of cycles	1520
Maternal age (years)	
<35	1009 (66.4%)
≥35	511 (33.6%)
Paternal age (years)	
<35	793 (52.2%)
≥35	727 (47.8%)
Infertility type	
Primary	704 (46.3%)
Secondary	816 (53.7%)
Infertility factors	
Tubal factor	780 (51.3%)
Ovulatory dysfunction	83 (5.5%)
Tubal factor and ovulatory dysfunction	4 (0.3%)
Other factors	653 (43.0%)
Fertilization methods	
IVF	1233 (81.1%)
ICSI	272 (17.9%)
IVF+ICSI	15 (1.0%)
COH protocol	
GnRH-a long protocol	795 (52.3%)
GnRH antagonist protocol	441 (29.0%)
Long term follicular protocol	113 (7.4%)
GnRH-a short protocol	120 (7.9%)
GnRH-a prolonged protocol	29 (1.9%)
Other protocol	22 (1.4%)
Forward motile sperm concentration after treatment (×10^6^/mL)	
<15	1070 (70.4%)
≥15	450 (29.6%)
Total Gn dose	3047.7 ± 1054.2
OHSS	
No	1504 (98.9%)
Yes	16 (1.1%)
Embryo transfer time	
Cleavage stage	1444 (95.0%)
Blastocyst stage	76 (5.0%)
Number of transferred embryos	
1	284 (18.7%)
2	1213 (79.8%)
3	23 (1.5%)
Hormone level on HCG day	
Estradiol (E_2_) (pg/ml)	2420.8 ± 1363.2
Progesterone (P) (ng/ml)	0.74 ± 0.47
Reproductive outcomes	
Clinical pregnancy	684 (45.0%)
Biochemical pregnancy	117 (7.7%)
Live birth	531 (34.9%)

COH, controlled ovarian hyperstimulation; OHSS, ovarian hyperstimulation syndrome; IVF, *in vitro* fertilization; ICSI, intracytoplasmic sperm injection.

### 3.2 Comparison of characteristics between patients with high and low E_2_

**Figure 1:**
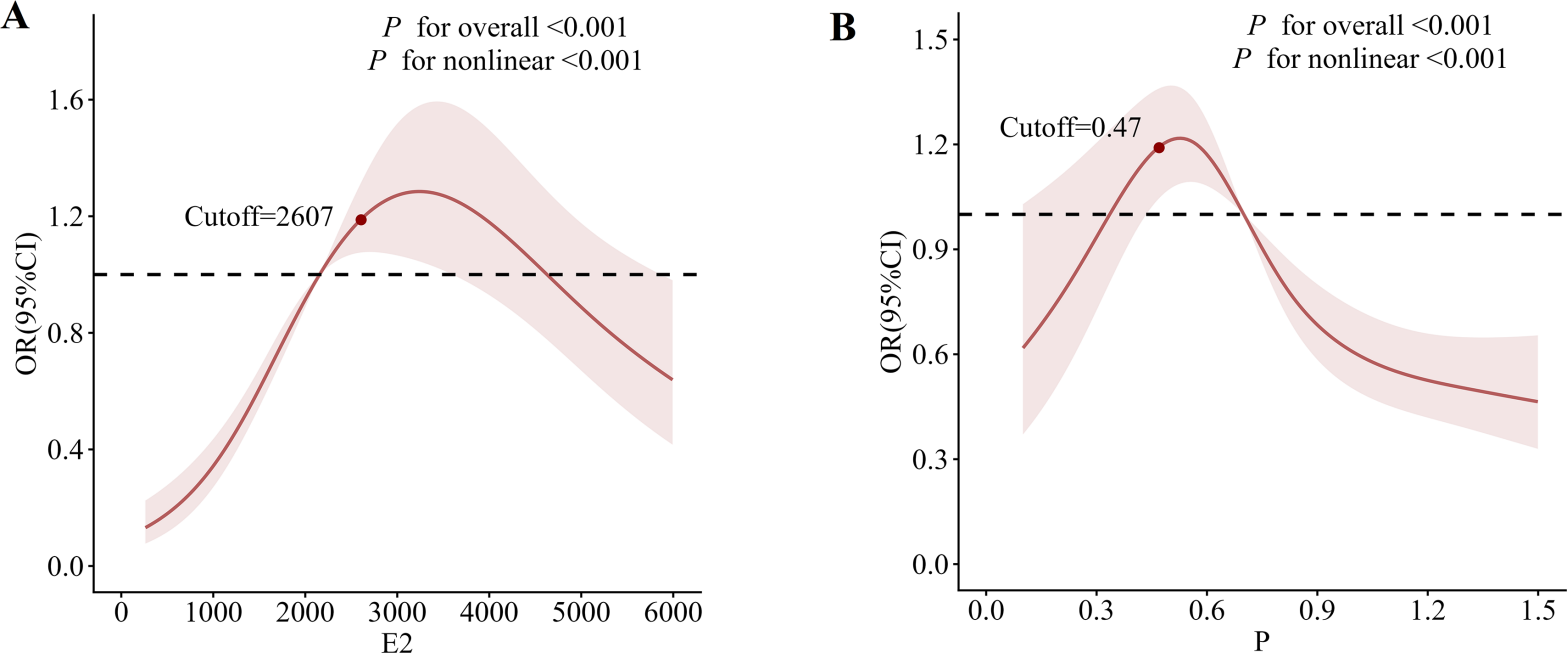
Relationship between E_2_ **(A)** and P **(B)** on HCG day and clinical pregnancy in a RCS model. Note: The red curve and red shaded area represent the odds ratio (OR) and 95% confidence interval (CI) for the predicted probability of clinical pregnancy, respectively. The slope of the curve changed significantly at E_2_ = 2607.0 pg/mL and P = 0.47 ng/mL on HCG day, which were defined as clinically meaningful cutoff values (*P* < 0.001). The RCS analysis showed a non−linear dose−response relationship between E_2_, P levels on HCG day and clinical pregnancy (*P* for non−linearity <0.001). RCS, restricted cubic spline; E_2_, estradiol; P, progesterone.

RCS analysis revealed that the cutoff values of E_2_ (**Figure 1A**) and P (**Figure 1B**) on HCG day was 2607.0 pg/mL and 0.47 ng/mL, respectively. There were 961 cycles with E_2_ ≤2607.0 pg/mL and 559 cycles with E_2_ >2607.0 pg/mL. Clinical pregnancy rate was higher in the cycles with E_2_ ≤2607.0 pg/mL (486/961, 50.6%) than that in the cycles with E_2_ >2607.0 pg/mL (198/559, 35.4%) (*p* < 0.001). Biochemical pregnancy rate was lower in the cycles with E_2_ ≤2607.0 pg/mL (60/961, 6.2%) than that in the cycles with E_2_ >2607.0 pg/mL (57/559, 10.2%) (*p* = 0.007). There was no relationship between maternal age, paternal age, infertility type, infertility factors, fertilization methods, forward motile sperm concentration after treatment, OHSS, live birth rate and the cycles with difference E_2_ levels (all *p*>0.05) (**Table 2**).

**Table 2:** Comparison of characteristics between patients with different E_2_ levels.

Variables	E_2_ ≤2607.0 pg/mL (n=961)	E_2_ >2607.0 pg/mL (n=559)	*p* values
Maternal age (years)			
<35	621 (64.6%)	388 (69.4%)	0.063 (χ^2^ = 3.633)
≥35	340 (35.4%)	171 (30.6%)	
Paternal age (years)			
<35	500 (52.0%)	293 (52.4%)	0.915 (χ^2^ = 0.021)
≥35	461 (48.0%)	266 (47.6%)	
Infertility type			
Primary	439 (45.7%)	265 (47.4%)	0.523 (χ^2^ = 0.423)
Secondary	522 (54.3%)	294 (52.6%)	
Infertility factors			
Tubal factor	503 (52.3%)	277 (49.6%)	0.361 (χ^2^ = 3.287)
Ovulatory dysfunction	57 (5.9%)	26 (4.7%)	
Tubal factor and ovulatory dysfunction	3 (0.3%)	1 (0.2%)	
Other factors	398 (41.4%)	255 (45.6%)	
Fertilization methods			
IVF	784 (81.6%)	449 (80.3%)	0.401 (χ^2^ = 1.899)
ICSI	170 (17.7%)	102 (18.2%)	
IVF+ICSI	7 (0.7%)	8 (1.4%)	
COH protocol			
GnRH-a long protocol	469 (48.8%)	326 (58.3%)	<0.001 (χ^2^ = 34.037)
GnRH antagonist protocol	323 (33.6%)	118 (21.1%)	
Long term follicular protocol	70 (7.3%)	43 (7.7%)	
GnRH-a short protocol	62 (6.5%)	58 (10.4%)	
GnRH-a prolonged protocol	21 (2.2%)	8 (1.4%)	
Other protocol	16 (1.7%)	6 (1.1%)	
Forward motile sperm concentration after treatment (×10^6^/mL)			
<15	675 (70.2%)	395 (70.7%)	0.907 (χ^2^ = 0.030)
≥15	286 (29.8%)	164 (29.3%)	
Total Gn dose	3125.0 (2475.0, 3825.0)	2625.0 (2100.0, 3300.0)	<0.001 (Z=-8.138)
OHSS			
No	954 (99.3%)	550 (98.4%)	0.121 (χ^2^ = 2.637)
Yes	7 (0.7%)	9 (1.6%)	
Reproductive outcomes			
Clinical pregnancy	486 (50.6%)	198 (35.4%)	<0.001 (χ^2^ = 32.783)
Biochemical pregnancy	60 (6.2%)	57 (10.2%)	0.007 (χ^2^ = 7.774)
Live birth	384 (40.0%)	147 (26.3%)	0.189 (χ^2^ = 1.843)

### 3.3 Comparison of characteristics between patients with high and low P

There were 394 cycles with P ≤0.47 ng/mL and 1126 cycles with P >0.47 ng/mL. Clinical pregnancy rate was higher in the cycles with P ≤0.47 ng/mL (219/394, 55.6%) than that in the cycles with P >0.47 ng/mL (465/1126, 41.3%) (*p* < 0.001). Biochemical pregnancy rate was lower in the cycles with P ≤0.47 ng/mL (17/394, 4.3%) than that in the cycles with P >0.47 ng/mL (100/1126, 8.9%) (*p* = 0.004). There was no relationship between maternal age, paternal age, infertility type, fertilization methods, forward motile sperm concentration after treatment, OHSS, live birth rate and the cycles with difference P levels (all *p*>0.05) (**Table 3**).

**Table 3:** Comparison of characteristics between patients with different P levels.

Variables	P ≤0.47 ng/mL (n=394)	P >0.47 ng/mL (n=1126)	*p* values
Maternal age (years)			
<35	248 (62.9%)	761 (67.6%)	0.095 (χ^2^ = 2.816)
≥35	146 (37.1%)	365 (32.4%)	
Paternal age (years)			
<35	209 (53.0%)	584 (51.9%)	0.725 (χ^2^ = 0.163)
≥35	185 (47.0%)	542 (48.1%)	
Infertility type			
Primary	168 (42.6%)	536 (47.6%)	0.100 (χ^2^ = 2.891)
Secondary	226 (57.4%)	590 (52.4%)	
Infertility factors			
Tubal factor	211 (53.6%)	569 (50.5%)	0.002 (χ^2^ = 14.457)
Ovulatory dysfunction	31 (7.9%)	52 (4.6%)	
Tubal factor and ovulatory dysfunction	3 (0.8%)	1 (0.1%)	
Other factors	149 (37.8%)	504 (44.8%)	
Fertilization methods			
IVF	326 (82.7%)	907 (80.6%)	0.396 (χ^2^ = 1.799)
ICSI	66 (16.8%)	206 (18.3%)	
IVF+ICSI	2 (0.5%)	13 (1.2%)	
COH protocol			
GnRH-a long protocol	155 (39.3%)	640 (56.8%)	<0.001 (χ^2^ = 53.531)
GnRH antagonist protocol	153 (38.8%)	288 (25.6%)	
Long term follicular protocol	48 (12.2%)	65 (5.8%)	
GnRH-a short protocol	24 (6.1%)	96 (8.5%)	
GnRH-a prolonged protocol	7 (1.8%)	22 (2.0%)	
Other protocol	7 (1.8%)	15 (1.3%)	
Forward motile sperm concentration after treatment (×10^6^/mL)			
<15	279 (70.8%)	791 (70.2%)	0.848 (χ^2^ = 0.044)
≥15	115 (29.2%)	335 (29.8%)	
Total Gn dose	2850.0 (2146.9, 3675.0)	3000.0 (2343.8, 3675.0)	0.107 (Z=-1.614)
OHSS			
No	391 (99.2%)	1113 (98.8%)	0.585 (χ^2^ = 0.433)
Yes	3 (0.8%)	13 (1.2%)	
Reproductive outcomes			
Clinical pregnancy	219 (55.6%)	465 (41.3%)	<0.001 (χ^2^ = 24.072)
Biochemical pregnancy	17 (4.3%)	100 (8.9%)	0.004 (χ^2^ = 8.566)
Live birth	167 (42.4%)	364 (32.3%)	0.556 (χ^2^ = 0.351)

### 3.4 Logistic regression analysis of the relationship of E_2_ and P levels on HCG day and clinical pregnancy, biochemical pregnancy

The results of univariate logistic analysis showed that E_2_ ≤2607.0 pg/mL on the HCG day (odds ratio (OR): 1.865, 95% confidence interval (CI): 1.505-2.312, *p* < 0.001), and P ≤0.47 ng/mL on the HCG day (OR: 1.779, 95% CI: 1.411-2.242, *p* < 0.001) increased the clinical pregnancy rate. The results of multiple logistic regression analysis showed that E_2_ ≤2607.0 pg/mL on the HCG day (OR: 2.040, 95% CI: 1.627-2.558, *p* < 0.001), and P ≤0.47 ng/mL on the HCG day (OR: 1.970, 95% CI: 1.539-2.521, *p* < 0.001) increased the clinical pregnancy rate adjusted maternal age, paternal age, infertility type, infertility factors, fertilization methods, COH protocol, forward motile sperm concentration after treatment, and OHSS.

The results of univariate logistic analysis showed that E_2_ ≤2607.0 pg/mL on the HCG day (OR: 0.586, 95% CI: 0.402-0.856, *p* = 0.006), and P ≤0.47 ng/mL on the HCG day (OR: 0.463, 95% CI: 0.273-0.784, *p* = 0.004) decreased the biochemical pregnancy rate. The results of multiple logistic regression analysis showed that E_2_ ≤2607.0 pg/mL on the HCG day (OR: 0.573, 95% CI: 0.387-0.848, *p* = 0.005), and P ≤0.47 ng/mL on the HCG day (OR: 0.455, 95% CI: 0.265-0.781, *p* = 0.004) decreased the biochemical pregnancy rate adjusted maternal age, paternal age, infertility type, infertility factors, fertilization methods, COH protocol, forward motile sperm concentration after treatment, OHSS, embryo transfer time, and number of transferred embryos (**Table 4**). In addition, multiple logistic regression analysis showed no statistically significant relationship between E_2_, P and live birth (**Table 4**).

**Table 4:** Logistic regression analysis of the relationship of E_2_ and P levels on HCG day and clinical pregnancy, biochemical pregnancy, and live birth.

Variables	Clinical pregnancy				Biochemical pregnancy				Live birth			
	Crude OR (95% CI)	*p* values	Adjusted OR (95% CI)	*p* values	Crude OR (95% CI)	*p* values	Adjusted OR (95% CI)	*p* values	Crude OR (95% CI)	*p* values	Adjusted OR (95% CI)	*p* values
E_2_ level on HCG day												
>2607.0 pg/ml	1.000 (reference)		1.000 (reference)		1.000 (reference)		1.000 (reference)		1.000 (reference)		1.000 (reference)	
≤2607.0 pg/ml	1.865 (1.505-2.312)	<0.001	2.040 (1.627-2.558)	<0.001	0.586 (0.402-0.856)	0.006	0.573 (0.387-0.848)	0.005	1.306 (0.888-1.922)	0.175	1.341 (0.885-2.032)	0.166
P level on HCG day												
>0.47 ng/ml	1.000 (reference)		1.000 (reference)		1.000 (reference)		1.000 (reference)		1.000 (reference)		1.000 (reference)	
≤0.47 ng/ml	1.779 (1.411-2.242)	<0.001	1.970 (1.539-2.521)	<0.001	0.463 (0.273-0.784)	0.004	0.455 (0.265-0.781)	0.004	0.891 (0.609-1.305)	0.554	0.857 (0.570-1.290)	0.460

OR, odds ratio; CI, confidence interval.

Adjust for: maternal age, paternal age, infertility types, infertility factors, fertilization methods, COH protocol, forward motile sperm concentration after treatment, OHSS, embryo transfer time, and number of transferred embryos.

### 3.5 Evaluation of the performance of E2 and P in pregnancy outcomes based on ROC analysis

**Figure 2:**
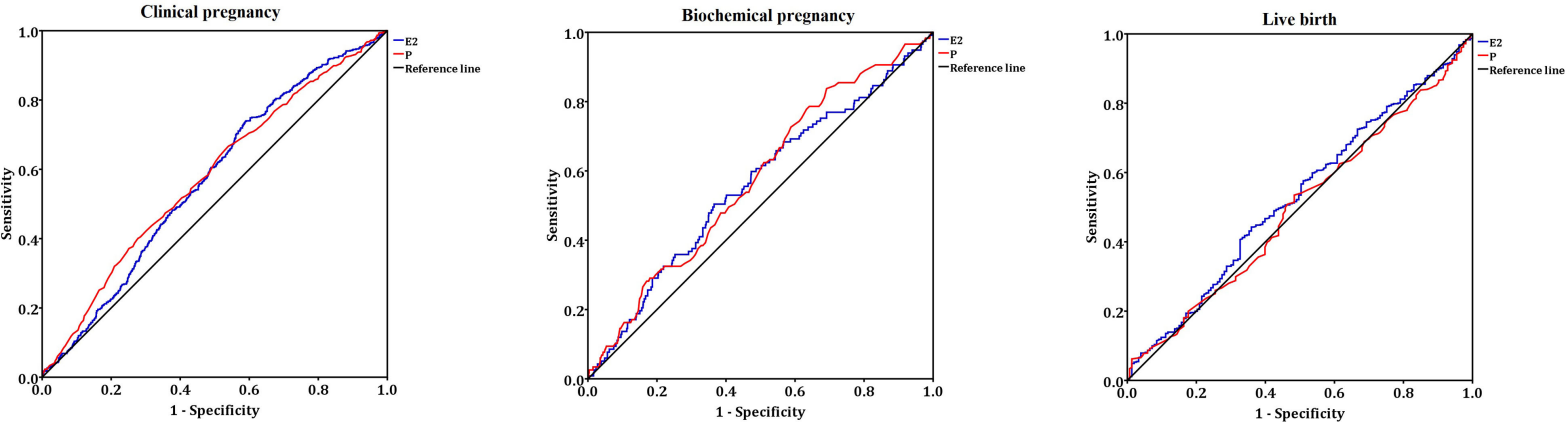
ROC analysis of E_2_ and P used in the prediction of pregnancy outcomes, such as clinical pregnancy, biochemical pregnancy, and live birth.

ROC analysis revealed that the areas under the ROC curves (AUC) of E_2_ for evaluating clinical pregnancy, biochemical pregnancy, and live birth were 0.570, 0.560, and 0.528, respectively. The sensitivity and specificity of E_2_ ≤2607.0 pg/mL for evaluating clinical pregnancy, biochemical pregnancy, and live birth were as follows: clinical pregnancy (sensitivity = 70.9%, specificity = 43.2%), biochemical pregnancy (sensitivity = 48.7%, specificity = 64.1%), and live birth (sensitivity = 72.1%, specificity = 33.3%) (**Figure 2**).

The areas under the ROC curves (AUC) of P for evaluating clinical pregnancy, biochemical pregnancy, and live birth were 0.579, 0.576, and 0.498, respectively. The sensitivity and specificity of P ≤0.47 ng/mL for evaluating clinical pregnancy, biochemical pregnancy, and live birth were as follows: clinical pregnancy (sensitivity = 30.3%, specificity = 79.8%), biochemical pregnancy (sensitivity = 85.5%, specificity = 25.6%), and live birth (sensitivity = 29.9%, specificity = 68.6%) (**Figure 2**).

## 4 Discussion

In this study, E_2_ and P levels on the HCG day within the identified threshold range increased the clinical pregnancy rate and decreased the biochemical pregnancy rate in fresh embryo transfer in IVF/ICS cycles. The clinical pregnancy rate, as the key indicator for evaluating the efficacy of ART, comprehensively reflects the quality of embryos, the receptivity of the endometrium, and the coordination of the hormonal environment (37, 38). The core feature of biochemical pregnancy is that the embryo stops developing early after implantation, and pregnancy can only be confirmed through blood HCG testing, while no gestational sac can be observed by ultrasound (39, 40). Its occurrence is closely related to the stability of embryo implantation and the developmental potential of the embryo. Notably, although no statistically significant associations were observed between hCG-day E_2_/P levels and live birth outcomes (*p* = 0.189 and *p* = 0.556), these p-values do not represent a complete lack of correlation but suggest marginal statistical trends. Such non-significant trends may be attributable to the inherent limitations of this retrospective single-center cohort, including potential insufficient statistical power and unmeasured residual confounding. In this study, E_2_ and P levels exhibited distinct associations across different pregnancy endpoints, with significant correlations observed for biochemical and clinical pregnancy but not for live birth.

The physiological function of E_2_ is to thicken the endometrium, preparing it for the implantation of the embryo and providing a stable uterine environment for the embryo’s growth and development (41, 42). From the perspective of E_2_ receptivity, a low E_2_ environment is more conducive to maintaining the synchronous proliferation and secretion of the endometrium (43). High E_2_ levels often lead to excessive endometrial proliferation, resulting in excessive glandular density and interstitial edema, which in turn disrupts the three-dimensional structure and cell communication microenvironment of the endometrium (44). Some studies suggested that excessively high serum E_2_ level on the HCG day had a negative impact on pregnancy outcomes (45–47). However, many studies suggest that the level of E_2_ has no impact on pregnancy outcomes. A meta-analysis showed that there was insufficient evidence to support a correlation between HCG-day serum estradiol and pregnancy rate (48). Michael et al. (49) conducted a retrospective study which indicated that an increase in estradiol on HCG day had no negative effect on live birth after controlling for embryo quality. A series of studies found that there was no correlation between estradiol concentration and successful pregnancy (50–52).

In addition, the results of this study confirm that the P level on HCG day was associated with clinical pregnancy rate (53, 54). The pregnancy rate of cycles with P ≤0.47 ng/mL (55.6%) was significantly higher than that of the P >0.47 ng/mL group (41.3%), and it is consistent with that of most studies (17, 55, 56). A study found that an increase in serum progesterone levels on the day of HCG administration was significantly associated with a decrease in pregnancy rate and implantation rate (55). The research conducted by Merit et al. has confirmed that the progesterone level in the late stage of follicles has a negative correlation with the reproductive outcomes of patients who receive exogenous gonadotropin ovarian stimulation (56). A prospective study found that an increase in P levels on the HCG day led to the early transformation of the endometrium into a secretory endometrium, which had an adverse effect on the pregnancy outcome (57). A meta-analysis study supported the evidence that in controlled ovarian stimulation cycles, an increase in HCG day P levels reduced the pregnancy rate; conversely, this effect did not exist in the thawing cycle (58).

Before HCG injection, the ovaries are in the follicular phase. Excessive P levels will activate the luteinizing hormone receptors (LHR) in the granulosa cells, causing the follicles to enter the luteal phase prematurely (59, 60). It leads to problems such as arrested maturation of the oocyte and hardening of the zona pellucida. In contrast, the follicular granulosa cells of patients with appropriate P level remain in an actively proliferative phase, ensuring the stable synthesis of estrogen within the follicles and the normal meiotic division of the oocyte. At the level of E_2_ regulation, appropriate P level can precisely control the opening time of the endometrial implantation window. An early increase in P will induce the endometrium to enter the secretory phase prematurely, resulting in the implantation window being closed when the embryo reaches the uterus. In contrast, in the appropriate P group, the endometrium will, after HCG injection, synchronously enter the secretory phase under the effect of P supplementation therapy, achieving a precise match between the embryo development stage and the endometrial receptivity stage (16, 61).

From the perspective of the coordination mechanism, appropriate E_2_ ensures follicle development, while appropriate P prevents premature luteinization. Together, they ensure the quality of the egg and the potential for embryo development. At the same time, appropriate E_2_ maintains the moderate proliferation of the endometrium, and appropriate P delays the transformation from endometrial secretion to secretion conversion. Together, they achieve synchronous development of the endometrium and the embryo. This coordination effect forms the optimal transplantation conditions for high-quality embryos and suitable endometrium.

Based on the results of this study, the following clinical practice recommendations are proposed for IVF/ICSI fresh embryo transfer cycles: First, patients with relatively optimal E_2_ and P levels on the HCG day can be prioritized for fresh embryo transfer to enhance the pregnancy success rate. Second, for patients with a high P level, it is recommended to conduct a comprehensive assessment of the embryo quality and the thickness of the endometrium and then consider attempting fresh embryo transfer. Otherwise, the fresh cycle should be cancelled and frozen-thawed embryo transfer should be chosen.

Although this study clearly demonstrated the association between low E_2_ and P levels and an increase in clinical pregnancy rate, there are still some limitations. Firstly, as a retrospective analysis, some clinical data were incomplete. Detailed embryo grading information and systematic records of endometrial parameters were unavailable, leading to potential residual confounding. Therefore, our results should be interpreted cautiously, and prospective studies with complete collection of the above variables are needed for further validation. Secondly, there were differences in the COH protocols among groups. Although all COH protocols were included as covariates in the multivariate logistic regression model and fully adjusted to minimize the influence of confounding factors, residual confounding could not be completely eliminated due to the heterogeneity of COH protocols. Moreover, the incidence rates of clinical pregnancy (45.0%) and live birth (34.9%) were both higher than 10%. Under such conditions, the odds ratios derived from logistic regression may overestimate the actual relative risk and cannot be directly approximated to risk ratios. For consistency with mainstream analytical and reporting conventions in reproductive medicine, we retained OR values for result presentation. However, this limitation should be noted when interpreting the effect size; the reported ORs should be interpreted prudently and not equated directly with risk ratios in clinical practice. Thirdly, this study only focused on the hormone levels on the HCG day and did not analyze the impact of the dose and duration of P supplementation therapy before transplantation on the outcome. Future studies can optimize the hormone support plan for patients with low P level after transplantation through randomized controlled trials. Finally, despite stable core protocols, potential minor temporal variations in patient characteristics or clinical management could not be entirely excluded, which may represent residual confounding.

This study investigated the association between serum E_2_, P levels on the day of HCG trigger and clinical pregnancy, biochemical pregnancy in IVF/ICSI fresh embryo transfer cycles. The main strength lies in its analysis based on real-world clinical data, which can provide practical reference for evaluating the relationship of embryo transfer timing, endocrine status and clinical pregnancy, biochemical pregnancy, and help optimize clinical management strategies for fresh embryo transfer cycles. Given the inherent limitations of a retrospective study, large-sample prospective cohort studies are recommended in the future to further clarify the objective impact of E_2_ and P levels on the day of HCG trigger on clinical pregnancy and biochemical pregnancy, and to provide higher-level evidence-based medical evidence for clinical practice.

## 5 Conclusions

In conclusion, the present study demonstrated that appropriate serum E_2_ and P levels on HCG day were significantly associated with favorable pregnancy outcomes in IVF/ICSI fresh embryo transfer cycles. Specifically, E_2_ ≤2607.0 pg/mL and P ≤0.47 ng/mL on HCG day were closely correlated with a significantly higher clinical pregnancy rate and a remarkably lower biochemical pregnancy rate. These findings providing a reference for endocrine monitoring and clinical decision−making during IVF/ICSI fresh embryo transfer cycles. Future prospective, multi−center studies with larger sample sizes are warranted to verify our results, optimize the endocrine monitoring criteria on HCG day, and explore more reliable predictors for pregnancy outcomes in fresh embryo transfer cycles. However, it should be emphasized that the serum E_2_ and P levels on the day of hCG administration are merely auxiliary clinical reference indicators rather than independent predictive biomarkers.

## Data Availability

The original contributions presented in the study are included in the article/supplementary material. Further inquiries can be directed to the corresponding author.
